# Quantitative susceptibility mapping of the brain is associated with inflammatory changes in Alzheimer’s disease related areas

**DOI:** 10.1177/0271678X261417193

**Published:** 2026-02-08

**Authors:** Seyyed Ali Hosseini, Stijn Servaes, Arthur C Macedo, Etienne Aumont, Nesrine Rahmouni, Tevy Chan, Joseph Therriault, Lydia Trudel, Brandon Hall, Yi-Ting Wang, Jaime Fernandez Arias, Gleb Bezgin, Yansheng Zheng, Marina P Gonçalves, Kely Quispialaya Socualaya, Marcel S Woo, Cécile Tissot, Delphine Oliva-Lopez, Jieying Li, Stuart Mitchell, Aurélie Lebrun, Robert Hopewell, Sanjeev Chawla, Vladimir Fonov, Gassan Massarweh, Yasser Iturria Medina, Jean-Paul Soucy, Maxime Montembeault, Paolo Vitali, Kaj Blennow, Thomas K Karikari, Andréa L Benedet, Nicholas J Ashton, Henrik Zetterberg, Tharick A Pascoal, Serge Gauthier, Jesse Klostranec, Hangwei Zhuang, Junghun Cho, D Louis Collins, Yi Wang, David A Rudko, Pedro Rosa-Neto

**Affiliations:** 1 Translational Neuroimaging Laboratory, McConnell Brain Imaging Centre, Montreal Neurological Institute, McGill University, Montreal, QC, Canada; 2 Department of Neurology and Neurosurgery, McGill University, Montreal, QC, Canada; 3 The McGill University Research Center for Studies in Aging, Douglas Hospital Research Centre, Centre Intégré Universitaire de Santé et Services Sociaux de l’Ouest-de-l’Île-de-Montréal, Verdun, QC, Canada; 4 Translational Neurodegeneration Laboratory, Department of Neurology, University Medical Center Hamburg–Eppendorf, Hamburg, Germany; 5 Institute of Neuroimmunology and Multiple Sclerosis, University Medical Center Hamburg–Eppendorf, Hamburg, Germany; 6 Lawrence Berkeley National Laboratory, Berkeley, CA, USA; 7 Department of Radiology, Perelman School of Medicine at the University of Pennsylvania, Philadelphia, PA, USA; 8 Department of Psychiatry and Neurochemistry, Institute of Neuroscience and Physiology, The Sahlgrenska Academy, University of Gothenburg, Mölndal, Sweden; 9 Clinical Neurochemistry Laboratory, Sahlgrenska University Hospital, Mölndal, Sweden; 10 Paris Brain Institute, ICM, Pitié-Salpêtrière Hospital, Sorbonne University, Paris, France; 11 Division of Life Sciences and Medicine, Neurodegenerative Disorder Research Center, University of Science and Technology of China, Hefei, P.R. China; 12 Department of Neurology, Institute on Aging and Brain Disorders, First Affiliated Hospital of USTC, University of Science and Technology of China, Hefei, P.R. China; 13 Department of Neurology, School of Medicine, University of Pittsburgh, Pittsburgh, PA, USA; 14 Wallenberg Centre for Molecular Medicine, University of Gothenburg, Gothenburg, Sweden; 15 King’s College London, Institute of Psychiatry, Psychology and Neuroscience, Maurice Wohl Institute Clinical Neuroscience Institute, London, UK; 16 NIHR Biomedical Research Centre for Mental Health and Biomedical Research Unit for Dementia at South London and Maudsley NHS Foundation, London, UK; 17 Department of Neurodegenerative Disease, UCL Institute of Neurology, Queen Square, London, UK; 18 UK Dementia Research Institute at UCL, London, UK; 19 Department of Pathology and Laboratory Medicine, University of Wisconsin School of Medicine and Public Health, Madison, WI, USA; 20 Wisconsin Alzheimer’s Disease Research Center, University of Wisconsin School of Medicine and Public Health, University of Wisconsin–Madison, Madison, WI, USA; 21 Hong Kong Center for Neurodegenerative Diseases, InnoHK, Hong Kong, China; 22 Centre for Brain Research, Indian Institute of Science, Bangalore, India; 23 Department Psychiatry, School of Medicine, University of Pittsburgh, Pittsburgh, PA, USA; 24 Department of Diagnostic and Interventional Neuroradiology, Montreal Neurological Institute and Hospital, McGill University, Montreal, QC, Canada; 25 Department of Radiology, Weill Cornel Medicine, New York, NY, USA; 26 Department of Biomedical Engineering, the George Washington University Washington, D.C., USA; 27 Department of Biomedical Engineering, McGill University, Montreal, QC, Canada; 28 The Peter O’Donnell Jr. Brain Institute (OBI), University of Texas Southwestern Medical Centre (UTSW), Dallas, TX, USA

**Keywords:** QSM, brain’ susceptibility, neuroinflammation, Alzheimer’s disease, immune biomarkers

## Abstract

Accumulation of paramagnetic substances in brain tissue may constitute a feature of Alzheimer’s disease (AD) associated with inflammatory processes. This study employed MRI quantitative susceptibility mapping (QSM), as an index of paramagnetic load, to assess its association with brain Aβ and tau aggregates, as well as inflammatory biomarkers. We assessed QSM and T1-weighted MRI scans from 315 participants in the TRIAD cohort, including young-controls and individuals across the AD spectrum. Imaging was performed at baseline, with follow-up assessments at 12 and 24 months. Mean-cortical and subcortical susceptibility values were measured, and correlations with AD-relevant plasma and CSF inflammatory biomarkers. At baseline, AD patients had significantly greater QSM than age-matched controls in the posterior cingulate cortex, precuneus, and basal ganglia. After 24 months, QSM increased in the anterior cingulate in MCI, while dementia cases showed increase in the pallidum and hippocampus. Multiple comparison analysis indicated correlation between QSM and immune biomarkers IL-10RB, PD-L1, SCF, TWEAK, CSF-1, CXCL9, HGF, and CD40, but not with brain Aβ or tau-related biomarkers. Our findings reveal that the magnitude of tissue susceptibility load, as measured by QSM, reflects tissue inflammation rather than protein aggregation. QSM provides new insights into tissue dysfunction, with potential applications in AD therapeutic development.

## Introduction

Alzheimer’s disease (AD) is increasingly recognized as a neurodegenerative disorder with a strong immunopathological component. Protein aggregates formed by misfolded proteins as amyloid-β (Aβ) plaques and tau neurofibrillary tangles, can trigger innate immune responses in the brain by microglia and astrocyte activation.^1^ Given the clinical importance of neuroinflammation in AD, there is considerable interest in identifying imaging biomarkers for activated glia in vivo as a means of monitoring disease progression and measuring therapeutic interventions.

Quantitative susceptibility mapping (QSM) is a promising magnetic resonance imaging (MRI) technique to fill this gap by quantitatively detecting biophysical tissue alterations of AD pathology.^2–4^ QSM quantifies tissue magnetic susceptibility that reflects molecular electron composition.^5^ In practice, QSM is extremely sensitive to paramagnetic substances such as tissue iron and blood degradation products, with a significant impact on local susceptibility signals.^6^ Although the susceptibility is not molecule-specific, positive QSM value is generally interpreted as an iron deposition proxy in neurodegenerative disease research.^7^ QSM has been sensitive to detect the effect of the iron chelator Deferiprone in AD.^8^ This is especially relevant to AD, where disrupted iron homeostasis and focal iron deposition are well-documented pathological features.^9^ Imaging and *post-mortem* studies have demonstrated excess iron in AD-vulnerable regions, co-localized with Aβ plaques and tau tangles.^4,10^ Iron accumulation and neuroinflammation appear to influence each other in a reinforcing manner, where oxidative stress and microglial activation can co-occur,^10,11^ and evolve together over disease progression.^12^ Activated microglia can sequester and redistribute iron while iron induces oxidative stress and a pro-inflammatory microglia phenotype,^13^ reflecting the close interplay between iron handling and inflammatory signaling in AD,^14^ and recently are established biomarkers for active inflammatory processes in neuroimmunological diseases like multiple sclerosis.^15^ In this environment, the pathological susceptibility signals identified by QSM may show inflammation-associated iron metabolism in the brain. QSM-derived susceptibility increases are therefore interpreted as reflecting iron-related tissue changes that occur alongside neuroinflammatory processes, rather than serving as a direct measure of glial activation.^4^ It is important to note that QSM reflects bulk magnetic susceptibility and can also be influenced by tissue myelin content, vascular oxygenation, and mineral deposition; therefore, changes in susceptibility are interpreted within the broader context of regional anatomy and neurodegenerative progression.^16^

Several recent studies support the application of QSM in detecting AD-related brain changes and promote its utility as a clinical biomarker.^4,17^ Cross-sectional imaging studies have revealed that AD patients exhibit significantly higher magnetic susceptibility in some brain regions, including hippocampus, and basal ganglia, than age-matched controls. These regional QSM increases follow known loci of AD pathophysiology and associate with quantification of disease severity.^18,19^ Elevated QSM values in AD brain are linked with greater Aβ plaque burden and reduced cognitive performance, suggesting association between tissue susceptibility change and concomitant neuropathological burden.^19^ As such, greater baseline QSM in the hippocampus was a univariate predictor of increased cognitive decline in one group of subjects with preclinical Aβ deposition, highlighting the clinical predictive significance of susceptibility imaging.^19^ While this advancement has been reached, pathophysiologic explanation of AD QSM alterations is not yet appreciated to its fullest extent. QSM is traditionally interpreted to stand for iron/mineral accumulation, secondary to amyloid and tau pathology, but it is not clear how extensively these susceptibility alterations also indicate the inflammatory processes with which AD pathology is entangled.^4^

As such, the present study sought to assess the association between QSM and neuroinflammation biomarkers. We specifically investigate the correlations between magnetic susceptibility values derived with QSM and inflammatory changes through the Lense of various plasma and cerebrospinal fluid (CSF) biomarkers.

## Methods

### Participants

In this study, we initially screened 385 participants from the Translational Biomarkers in Aging and Dementia (TRIAD) cohort at McGill University.^20^ Exclusion criteria included non-AD dementia diagnoses, such as frontotemporal dementia or MCI not due to AD.^21^ To minimize confounding effects from microvascular lesions, susceptibility-weighted imaging (SWI) scans were reviewed during quality control, and participants with microbleeds in regions of interest were systematically excluded. This resulted in the exclusion of 70 individuals, leaving 315 subjects in the baseline cohort. Participants classified as cognitively unimpaired (CU) had no signs of cognitive impairment and a Clinical Dementia Rating (CDR) of 0. Participants with mild cognitive impairment (MCI) had a CDR of 0.5, indicating mild objective cognitive impairment but relative preservation of daily activities.^22^ Participants diagnosed with AD dementia had a CDR of one or two and met the diagnostic criteria for probable AD.^23^ A multidisciplinary panel of neurologists, neuropsychologists, and neuroscientists staged individuals as MCI or AD dementia patients, based on NIA-AA criteria for MCI due to AD^24^ and AD dementia, respectively.^25^ Additionally, participants with contraindications to MRI or PET imaging (such as metal implants, claustrophobia, or radiation sensitivity) were systematically excluded before screening. The study was approved by the Montreal Neurological Institute Working Committee and the Douglas Mental Health University Institute Research Ethics Board (IUSMD 16-60) and was conducted in accordance with the Declaration of Helsinki. Informed consent was obtained from all participants after a complete disclosure of research procedures.

### Biomarker measurements

A subset of participants had both plasma and CSF biomarkers measured, including phosphorylated tau (P-tau) isoforms (181, 217, and 231) and Aβ (40, 42), which were assessed with Simoa, ELISA, or ECLIA assays depending on platform availability, along with various markers of immune response and neuroinflammation measured using the Olink^®^ Target 96 Inflammation panel. The assay details are provided in Supplementary Information, Section 1. Cognitive function was also assessed in most participants (94%) using standardized protocols. The details of these tests have been described previously.^20,26,27^

### PET data acquisition

All participants underwent [^18^F]AZD4694 amyloid-PET and [^18^F]MK6240 tau-PET imaging performed on the same brain-dedicated scanner (Siemens high-resolution research tomograph, HRRT). The details of PET data acquisition are provided in Supplementary Information, Section 2.

### MRI data acquisition

**Figure 1.**
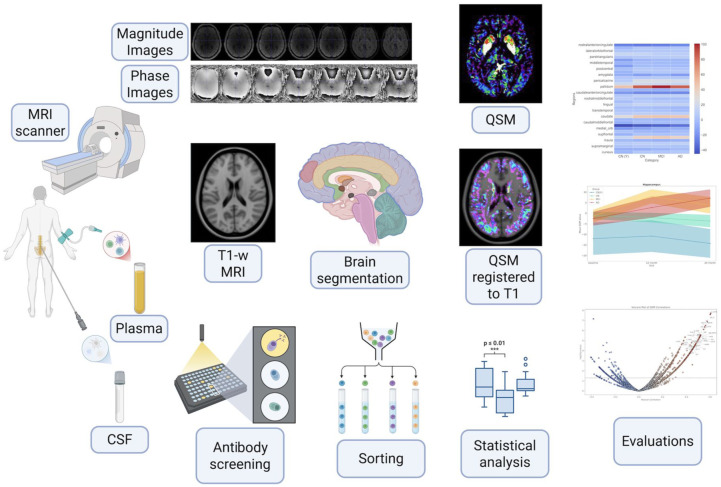
The full study schema. Figure 1 was created in BioRender (https://BioRender.com/r61a349).

A 3 Tesla Siemens MAGNETOM MRI scanner equipped with a standard head coil was used to acquire T1-weighted and T2*-weighted multi-echo gradient-recalled echo (GRE) sequences. All MRI scans were acquired on the same 3 T scanner at a single site with automated B0 shimming and vendor-supplied field-map calibration performed prior to each session. Routine weekly phantom-based quality control ensured scanner stability across the study period. The T1-weighted image was acquired using a 3D Magnetization-Prepared Rapid Gradient Echo (MPRAGE) sequence with the following parameters: voxel size = 1.0 × 1.0 × 1.0 mm (isotropic), field of view (FOV) = 256 mm, repetition time (TR) = 2300 ms, echo time (TE) = 2.96 ms, flip angle = 9°, and no fat or water suppression. The T2*-weighted GRE sequence was acquired for QSM^28^ with the following parameters: voxel size = 1.0 × 1.0 × 1.0 mm (isotropic), field of view = 224 mm, slice thickness = 1.00 mm, TR = 40.0 ms, TEs = 7 echoes ranging from 5.00 to 35.00 ms, flip angle = 17°, and no fat or water suppression. Figure 1 shows the full study schema, describing the integration of phase and magnitude T2*-weighted GRE sequences as inputs of the pipeline to generate QSM maps, registration of QSM maps to their native T1-weighted images, transformation of the DKT atlas to T1 native space, and comparison of regional QSM values with various plasma and CSF biomarkers.

### QSM processing pipeline

QSM reconstruction was conducted using a standardized processing pipeline in MATLAB 2018, which included the following steps:

### Phase processing

A Laplacian-based phase unwrapping method was applied to correct phase aliasing artifacts, ensuring phase continuity and reducing noise-related artifacts.^29^ The unwrapped phase images from multiple echoes were combined using a complex weighted combination approach, improving the signal-to-noise ratio (SNR) for subsequent processing.^30^ To isolate the local tissue-induced magnetic field from background field contributions, the projection onto dipole fields (PDF) method was applied, which effectively eliminates background fields originating from susceptibility sources outside the brain region of interests.^31^ The ill-posed dipole inversion problem was solved using the Morphology Enabled Dipole Inversion (MEDI + 0) algorithm. MEDI + 0 incorporates structural information from magnitude images and CSF as zero reference in reconstructing the susceptibility map, improving the accuracy of the estimated susceptibility values.^32,33^ The CSF reference was defined individually for each participant in native space, which was critical to reduces inter-subject variability related to global CSF susceptibility differences and ensures consistency in relative susceptibility comparisons across individuals. QSM values are reported in parts per million (ppm) and represent relative susceptibility referenced to CSF using the MEDI + 0 uniform CSF zero-reference approach.

### QSM post-processing

The QSM maps were registered to their native T1-weighted image using an affine transformation in advanced normalization tools (ANTs). The Desikan–Killiany–Tourville (DKT) atlas,^34^ initially in Montreal Neurological Institute 152 (MNI) space,^35^ was then transformed non-linearly to each subject’s T1 native space using an inverse transformation matrix obtained from T1 to MNI registration. For ROI-based analysis, the registered DKT atlas in native T1 space was then applied to each QSM maps, in order to measure the mean susceptibility in each region. For the voxel-wise analysis QSM maps were linearly registered to the native T1-weighted image space, and the T1-weighted images were linearly and nonlinearly registered to the MNI standardized space. To reduce confounding influences from atrophy and mixed tissue composition, QSM values were extracted in native space using individualized DKT-based segmentations and interpreted in parallel with regional volume measures to help distinguish susceptibility differences driven by iron-related processes from those arising from structural loss or other susceptibility sources.

### Statistical analysis

All statistical analyses were conducted in Python 3.9.12 using in-house scripts. The comparison of participants based on their clinical diagnoses was conducted using analysis of covariance (ANCOVA), with age included as a covariate. Post-hoc pairwise comparisons were performed using Tukey’s honestly significant difference (HSD) test, and results were corrected for multiple comparisons across all ROIs, using the false discovery rate (FDR, *q* < 0.05). To graph relationships between variables, scatter plots were plotted with linear regression lines, *p* values, and Spearman correlation coefficients (rho), using Matplotlib and SciPy libraries. All *p* values across the panel of regional QSM versus biomarker correlations were corrected for multiple comparisons using the Benjamini–Hochberg FDR procedure, with a *q*-value threshold of 0.05. Across all region-biomarker combinations, a total of 5180 correlations (37 brain regions × 140 plasma biomarkers) were tested, and for the associations surviving FDR correction we report effect sizes (Spearman rho) and 95% confidence intervals (CI) using non-parametric bootstrapping (1000 iterations). The heatmap was generated using Seaborn’s clustermap function, and the hierarchical clustering was performed using the Ward method. Clusters were determined using Spearman correlation coefficients between QSM regions and various plasma/CSF biomarkers to detect distinctive patterns of susceptibility relationships. This method was implemented to capture the link between different brain QSM and other biomarkers.

For each of the QSM regions and four diagnostic groups, we fitted linear mixed-effects models with visit as a categorical fixed effect (0 = baseline, 1 = 12 months, 2 = 24 months) and a random intercept for each participant (ID). We used the formula QSM ~ C (Visit_Num) in Python with statsmodels toolbox. From each model we extracted the 12- and 24-month fixed-effect coefficients (β), their standard errors (SE), *p* values, and confirmed that all region-group models converged. To compare longitudinal changes between diagnostic groups, we modeled the difference in 12- and 24-month QSM changes for each region between AD versus CU, MCI versus CU, and CU (Y) versus CU. We fit models with time as a categorical fixed effect (baseline, 12, 24 months) fully interacted with diagnostic category using the formula QSM ~ C (time) × Category in Python (statsmodels), applying cluster-robust standard errors at the participant (ID) level. The interaction coefficients (e.g. C (time)[T.t12]:Category) represent the group-to-group differences in QSM change at each timepoint, from which we obtained the estimated differences, standard errors, and *p* values. QSM values were derived and analyzed in each participant’s native anatomical space, and longitudinal effects were estimated using within-subject mixed-effects models, which mitigate the influence of scanner drift and coil variability on susceptibility measurements.

Moreover, the volume of each brain region as delineated from registered DKT atlas to each native T1 image was compared to the mean QSM values extracted in those regions. Based on prior AD imaging and pathology studies, we designated the Posterior cingulate cortex, hippocampus, parahippocampal gyrus, insula, and precuneus as our primary regions of interest. These areas consistently show early neurodegenerative or functional-connectivity changes in AD.^36,37^ All other DKT atlas regions were analyzed in a secondary, hypothesis-generating framework.

Voxel-based linear regression analyses were performed to examine associations between QSM map and both CSF and plasma biomarkers, as well as between QSM map and amyloid/tau PET burden, using the MATLAB-based VoxelStats toolbox.^38^ All models were adjusted for age and sex. Multiple comparison correction was applied using random field theory,^39^ with a significance threshold of *p* < 0.001. The resulting statistical t-maps were visualized by overlaying them on a standardized structural template using BrainNet Viewer (version 1.63)^40^ within MATLAB (version 2018a).

## Results

The final sample size in the current study consisted of 315 participants, including 36 young individuals under 25 years old (CU(Y)), 185 CU older adults, 45 individuals with MCI, 27 individuals with typical AD dementia, and 22 individuals with atypical AD dementia. Out of 315, 167 returned for follow-ups over a period of up to 2 years. We found no significant differences in years of education or age. MCI and AD participants showed significantly lower mini-mental state examination (MMSE) and CSF Aβ42 and significantly higher phosphorylated tau (ptau)s. Table 1 summarizes the full demographics of participants included in the current study.

**Table 1.** The full demographics of participants enrolled in this study.

Characteristic	CU (Y)	CU	MCI	AD
Number	36	185	45	49
Mean age, years (SD)	22.55 (1.80)	69.28 (10.39)	71.86 (5.28)	65.30 (9.14)
Female, number (%)	22 (61.11)	119 (64.32)	25 (55.55)	28 (57.14)
Mean education, years (SD)	16.62 (1.43)	15.45 (3.65)	15.86 (4.13)	14.60 (3.34)
Mean MMSE score, (SD)	29.84 (0.43)	29.08 (1.03)	28 (2.02)	21.41 (5.40)
APOE ε4, number (%)	9 (22.22)	51 (27.56)	29 (64.44)	28 (57.14)
Total amyloid SUVR^a^, mean (SD)	1.19 (0.07)	1.46 (0.34)	2.36 (0.46)	2.33 (0.51)
Total tau SUVR^b^, mean (SD)	1.02 (0.10)	1.07 (0.17)	1.69 (0.60)	2.94 (1.26)
CSF Aβ 42, pg/ml, mean (SD)	783.04 (229.04)	991.43 (410.91)	577.11 (161.166)	449.94 (124.14)
Plasma ptau 181, mean (SD)	7.68 (3.62)	11.03 (7.09)	16.81 (6.37)	22.59 (10.39)
Plasma ptau 217, mean (SD)	0.03 (0.01)	0.06 (0.04)	0.16 (0.07)	0.28 (0.15)
Plasma ptau 231, mean (SD)	9.11 (5.99)	14.92 (8.84)	18.94 (9.29)	25.09 (10.57)

a Amyloid cutoff = 1.55 SUVR Neocortex.

b Tau cutoff = 1.18 SUVR MetaROI.

**Figure 2.**
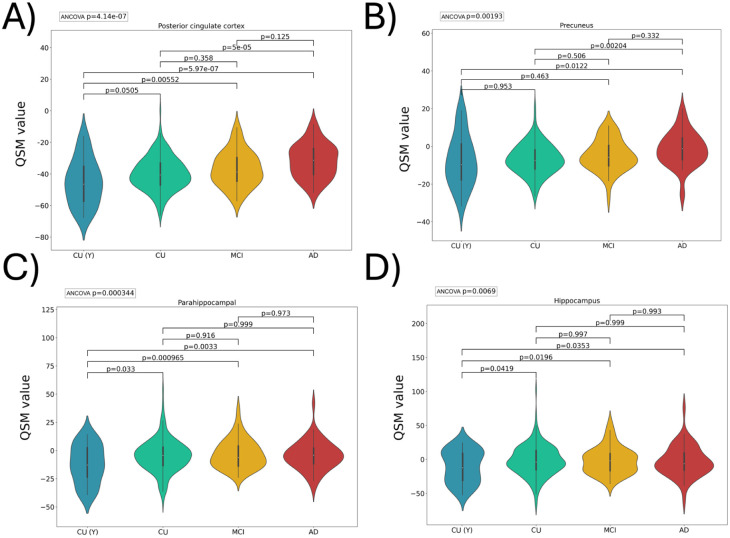
Regional QSM (values reported in ppm relative to CSF) differences across diagnostic groups were examined using ANCOVA, including age as a covariate. Post-hoc pairwise comparisons were conducted using Tukey’s HSD test. To control for type I error across multiple ROIs, *p* values were adjusted using the FDR method, with *q* < 0.05 considered statistically significant (only regions exhibiting significant ANCOVA *p* values are represented). Some brain region (a, b) showed significant differences due to AD (CU vs AD) and no region (c, d) showed significant differences between MCI versus AD or CU versus MCI. ANCOVA: analysis of covariance; HSD: honestly significant difference.

**Figure 3.**
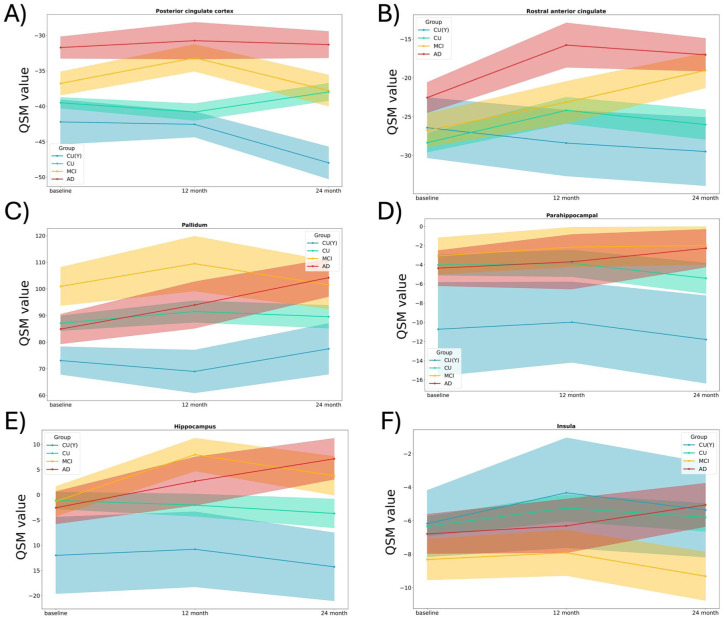
Longitudinal changes over 2-year follow-up. After 2 years of follow-up different brain regions (values reported in ppm) demonstrated distinct alteration in MCI and AD group comparing to CU (Y) and/or CU participants (a). Based on this figure, at least 2-year follow-up is warranted to capture the abnormal change of brain’s susceptibility due to AD. The MCI group exhibited increases only in the Rostral anterior cingulate (b; β = 7.79 ± 2.72; *p* < 0.001). Pallidum (c; β = 14.29 ± 5.80; *p* = 0.02) and hippocampus (e; β = 8.54 ± 3.18; *p* = 0.04) showed QSM increases only for the dementia group, whereas the CU (Y) and CU groups showed no changes during the same observation period (a–f). In complementary interaction analyses, limited group differences were detectable at 12 months: MCI showed a greater increase than CU in the hippocampus (β = 0.32 ± 0.11; *p* = 0.003), while CU (Y) differed from CU across several regions, including the pallidum (β = 0.41 ± 0.14; *p* = 0.004), posterior cingulate cortex (β = 0.27 ± 0.10; *p* = 0.009), precuneus (β = 0.25 ± 0.10; *p* = 0.014), hippocampus (β = 0.29 ± 0.10; *p* = 0.006), and parahippocampal gyrus (β = 0.31 ± 0.11; *p* = 0.005). No 12-month differences were observed between AD and CU. At 24 months, AD showed a significantly greater increase than CU in the hippocampus (β = 0.58 ± 0.19; *p* = 0.002), with no additional significant contrasts for MCI or CU (Y).

QSM values were predominantly negative in most brain regions, except in the basal ganglia, where the pallidum had the highest QSM values (Supplemental Figure 1). We observed distinct patterns of progression in QSM values over 24 months in CU and AD. At baseline, the posterior cingulate and precuneus showed a higher QSM load in AD versus CU. No region showed a significant difference between CU versus MCI, or MCI versus AD (Figure 2). We found that a 2-year follow-up was required to capture changes in QSM values across different brain regions (Figure 3). The MCI group exhibited increases only in the rostral anterior cingulate. Pallidum and hippocampus showed QSM increases only for the dementia group, whereas the CU (Y) and CU groups showed no changes during the same observation period. Notably, in the hippocampus, QSM values surged from an average of −2.5 ppm at baseline to over six ppm after 2 years in the dementia group. To assess whether shorter follow-up intervals could detect meaningful group differences, we directly compared 12- and 24-month QSM changes between diagnostic categories. At 12 months, only limited group differences were detectable. The MCI group showed a greater increase than CU in the hippocampus, while CU (Y) differed from CU across several regions, including the pallidum, posterior cingulate cortex, putamen, precentral cortex, caudate, precuneus, hippocampus, and parahippocampal gyrus. In contrast, no 12-month differences were observed between AD and CU. By 24 months, however, the AD group demonstrated a significantly greater QSM increase than CU in the hippocampus, indicating that diagnostically meaningful divergence in tissue susceptibility emerges more reliably over 2 years.

**Figure 4.**
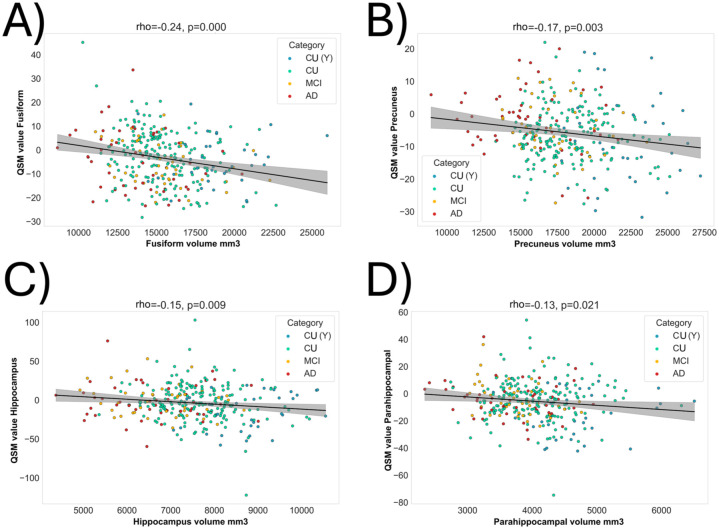
Stronger brain’s atrophy is correlated with higher brain’s magnetic susceptibility. Significant correlations were observed between the volume and QSM values (values reported in ppm) of various brain regions (a–d).

QSM values in various brain regions were negatively correlated with their region volume. This includes the hippocampal and parahippocampal regions, precuneus, and fusiform region, indicating a relationship between higher atrophy and increased susceptibility in AD-relevant regions of the brain (Figure 4).

**Figure 5.**
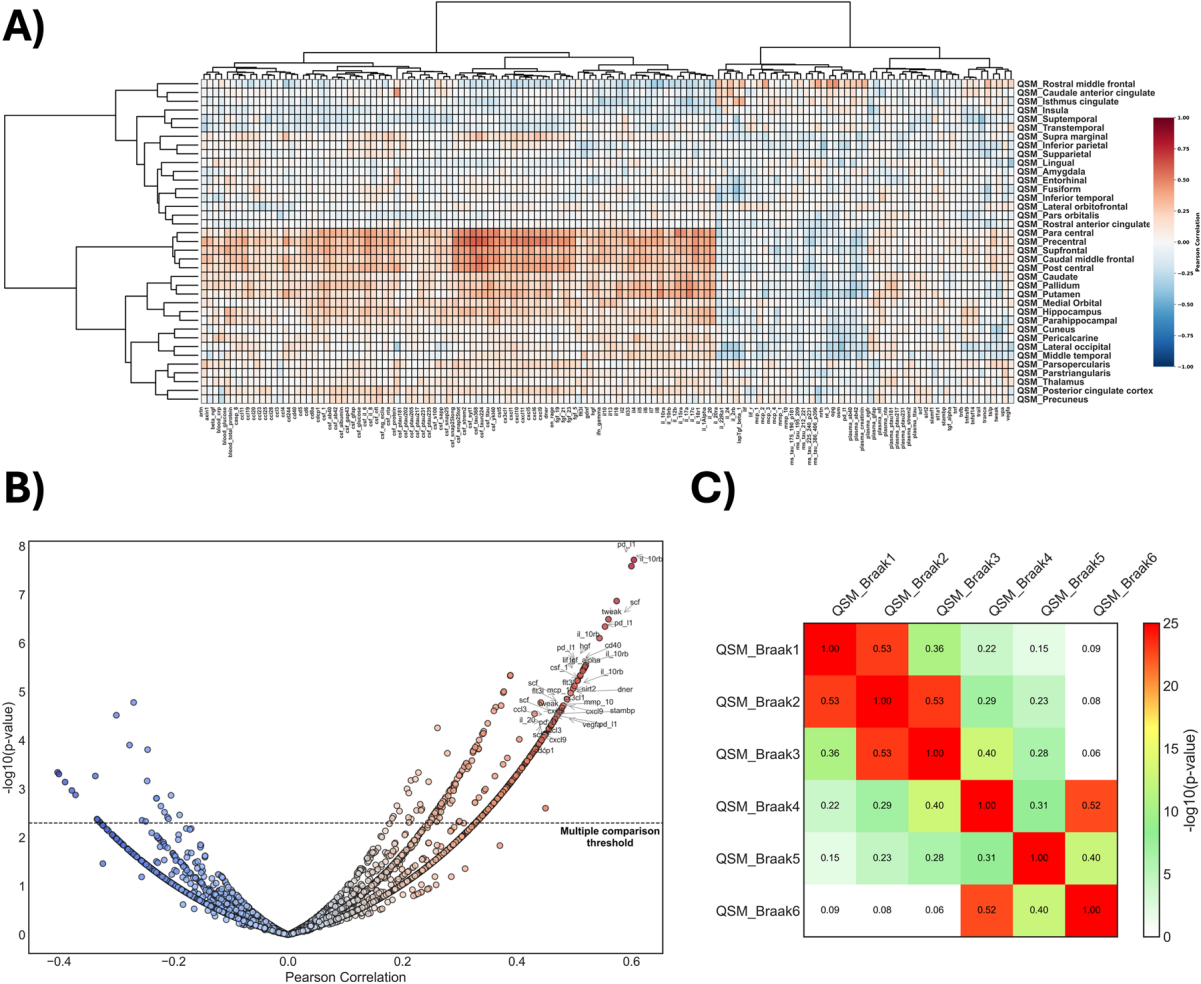
Associations between plasma immune markers and regional brain susceptibility (QSM). (a) Heatmap displaying Spearman correlation coefficients between plasma immune-related biomarkers and QSM values across different brain regions. Rows represent regions, and columns represent biomarkers, with hierarchical clustering applied to both axes. Warm colors indicate positive correlations, while cool colors indicate negative correlations. (b) Volcano plot showing the distribution of correlations between individual plasma biomarkers and regional mean QSM values. The x-axis shows the Spearman correlation coefficient, and the *y*-axis represents statistical significance as −log_10_ (*p* value). (c) correlation analysis across Braak stages revealed that the spatial distribution of QSM alterations aligns with brain regions typically implicated in Braak staging.

Clustering heatmap analysis was used to evaluate the correlation between QSM values in different brain regions and fluid biomarkers for Aβ, tau, and inflammation. We identified a distinct subset of immune biomarkers that exhibit multiple comparison corrected positive correlations with QSM values (Spearman rho >0.45, *q* < 0.01; Figure 5(a) and Supplemental Figure 2). Among these, interleukin-10 receptor subunit beta (IL-10RB; rho = 0.60, 95% CI: 0.45–0.76, *q* < 0.001), programmed cell death ligand 1 (PD-L1; rho = 0.59, 95% CI: 0.43–0.76, *q* < 0.001), stem cell factor (SCF; rho = 0.57, 95% CI: 0.44–0.71, *q* < 0.001), tumor necrosis factor-like weak inducer of apoptosis (TWEAK; rho = 0.55, 95% CI: 0.45–0.69, *q* < 0.001), colony stimulating factor 1 (CSF-1; rho = 0.51, 95% CI: 0.41–0.66, *q* < 0.001), C-X-C motif chemokine ligand 9 (CXCL9; rho = 0.49, 95% CI: 0.32–0.69, *q* < 0.001), hepatocyte growth factor (HGF; rho = 0.51, 95% CI: 0.34–0.69, *q* < 0.001), fms-like tyrosine kinase 3 ligand (FLT3L; rho = 0.49, 95% CI: 0.35–0.61, *q* < 0.001), cluster of differentiation 40 (CD40; rho = 0.52, 95% CI: 0.39–0.62, *q* < 0.001), stomatin-like protein 2 (STAMPB; rho = 0.47, 95% CI: 0.34–0.59, *q* < 0.001), and interleukin-20 (IL-20; rho = 0.45, 95% CI: 0.33–0.59, *q* < 0.001), emerged as top 11 biomarkers. Across AD-related regions, we observed no significant QSM correlations with CSF/plasma Aβ42/40, Aβ42, or p-tau-X (Figure 5(b)). Additionally, correlation analysis across PET Braak stages suggests that QSM distribution follows brain regions as predicted by the Braak pathological stages (Figure 5(c)), suggesting that magnetic susceptibility changes may reflect pathological processes that evolve in parallel with tau propagation.

**Figure 6.**
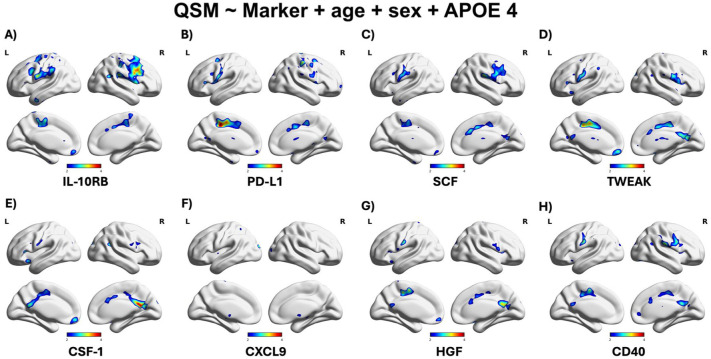
Voxel-based linear regression analyses identified cortical regions where the top 8 inflammation-related biomarkers showed the strongest associations with QSM measures (a–h). Warmer colors indicate higher t-values, highlighting areas linked to early (Braak I–II) and late (Braak V–VI) stages of AD pathology. All models were adjusted for age, sex, and APOE4 and significance was determined using random field theory correction (*p* < 0.001).

Voxel-based linear regression analyses in the elderly group (>65 years old) identified cortical regions where the top 11 inflammation-related markers exhibited the strongest associations (Figure 6). In contrast, no significant associations were observed between QSM measures and CSF Aβ40 or plasma p-tau isoforms (p-tau181, p-tau217, and p-tau231) in cortical areas (Supplemental Figure 3). Similarly, analyses examining the relationship between QSM maps and amyloid or tau PET burden revealed no cortical associations.

## Discussion

### Summary of findings

Based on our results, QSM revealed widespread regional increases in brain magnetic susceptibility in AD, with particularly elevated values in the basal ganglia and other AD-related regions. High QSM conveys the accumulation of paramagnetic biological products associated with disease pathophysiology. Our results support the growing framework linking QSM to neuronal injury, inflammation, and oxidative damage mediated by iron accumulation, with increased iron content providing a plausible explanation due to its role in microglial inflammatory responses. In the adult human brain, iron concentration in cortical gray matter can reach up to 200 μg/g, whereas it is typically lower in white matter (<60 μg/g).^41^ Approximately 90% of brain iron is stored as ferritin, with only 0.05% present in other forms.^42^ Iron homeostatic impairment induces oxidative stress via Fenton’s reaction, where iron interacts with reactive oxygen species (ROS) to produce highly reactive free radicals, which can, in turn, induce iron release from iron storage proteins and tissue damage and ferroptosis, which is an iron-dependent phospholipid peroxidation cell death.^43^ Our study sheds light on the interconnections between QSM and immune activation in AD.^4,14^ Additionally, we found that at least a 2-year follow-up is required to capture the overall change in QSM within the CI group.

### Reduced QSM values in brain diseases

QSM is a quantitative MRI technique that measures the magnetic susceptibility of tissues, which reflects their molecular composition. It enables non-invasive measurement of susceptibility sources in vivo, including iron.^4^ In QSM, paramagnetic substances (e.g. iron in ferritin or hemosiderin) increase the local magnetic field strength and supply positive susceptibility values, whereas diamagnetic substances (e.g. lipids, myelin, or calcium) yield negative susceptibility values because they weakly oppose the magnetic field.^44^ This means that iron-rich regions will be observed with higher (more positive) QSM values, and myelin-rich regions have lower (more negative) values. Altered brain iron metabolism has been linked to AD for decades. AD-related neurodegeneration is characterized by iron deposition and myelin loss, both of which are responsible for altering the balance of the paramagnetic and diamagnetic components of the brain.^45^ In line with our results, Au et al.^45^ demonstrated that the fimbria of the hippocampal white matter with high myelin density tends to have characteristically negative-susceptibility values in normal subjects, but in AD, it demonstrates a pattern toward positive values for QSM as demyelination and iron loading increase. In summary, some brain regions have negative values of QSM due to a predominance of diamagnetic tissue content (e.g. myelin or calcium excess), whereas others have positive values due to higher iron content of the paramagnetic type, which is a critical parameter, that is, dysregulated in dementia.^46^

### Regional susceptibility changes in AD

One of the common findings in QSM studies is that increased magnetic susceptibility is present in dementia patients in deep gray matter structures.^6,47^ The increases in QSM signal are assumed to reflect pathologic iron loading in the basal ganglia and limbic structures.^48,49^ A number of studies have shown that the susceptibility in dementia patients is significantly higher in the caudate nucleus, putamen, and amygdala.^50^ However, cortex, hippocampal, or thalamic QSM increases are not widely observed across studies.^51^ In comparison to our results, hippocampal or thalamic QSM did not show a significant difference in dementia patients compared to CU in the same age group. However, the posterior cingulate, superior temporal, precuneus, and inferior parietal regions showed marked differences between CU and dementia. There was no significant difference between CU versus MCI or between MCI versus AD. These variations are most likely a result of regional heterogeneity of AD or vascular abnormalities. Iron deposition is evident in certain regions associated with AD neuropathology, but other regions are less affected or show minor abnormalities. Disease stage is another critical factor: significant differences in QSM only become evident when pathology has reached a sufficiently advanced stage.^52^ Patients in early phases of the AD continuum (i.e. MCI), frequently exhibit no measurable increase in susceptibility as compared to healthy controls.^53^ Therefore, some areas (particularly those with strong iron affinity or dense pathology/plaque load) show significant QSM change in AD, while others do not, either because they are spared, or changes in these areas happen later or are too minor to matter in cross-sectional analysis.

### Longitudinal changes and the need for follow-up

AD pathology and its measurable impacts on QSM evolve gradually over time. Iron accumulation is a slow gradual process in the aging brain, accelerated in AD by chronic plaque deposition, tissue atrophy, and microhemorrhages.^54^ In the early clinical stages of the disease (MCI), susceptibility differences from normal aging can be very subtle. As described previously,^4^ MCI patients do not always demonstrate a significant QSM increase compared to controls, even in regions that will later go on to develop severe iron deposition. Therefore, a longitudinal approach with a sufficient follow-up period is necessary to reveal significant changes. In our study, a 2-year follow-up period was necessary to observe differences in QSM that were not apparent at baseline. Most AD patients progress from prodromal to more severe stages within a 2-year timeframe, during which iron likely continues to deposit in affected areas and myelin content may continue to decline. The cumulative effect over 24 months gives a measurable increase in magnetic susceptibility, that is, greater than inter-subject variability and scanner noise. Shorter durations (e.g. 6–12 months) may not capture these changes, as signal differences remain non-significant. By tracking the same individuals over 2 years, we increase sensitivity to change: each patient serves as their own control, so even modest increases in susceptibility become evident. This also aligns with evidence that QSM changes mirror disease progression; for example, patients progressing from MCI to AD dementia show new iron deposition imaged by follow-up QSM scans where baseline differences were not seen.^4^ However, clinical trials typically aim to quantify treatment-related effects rather than natural-history progression, and therapeutic modulation of iron or neuroinflammation may occur more rapidly than spontaneous disease-related QSM changes.

Therefore, the QSM alterations in AD as a whole (susceptibility increases in some regions) increase over time. Two years of follow-up might be sufficient to distinguish these evolving changes, so that susceptibility differences seen are truly due to disease progression and not to simple inter-individual statical differences. These longitudinal QSM data are valuable to comprehend the trajectory of AD: they demonstrate that iron dysregulation is a process that changes over time, and they reinforce the case for QSM as a biomarker to monitor disease progression or the impact of disease-modifying therapies.

### QSM as a marker of neuroinflammation and cytokine activity

Strong evidence associates iron deposition measured by QSM with mechanisms of neuroinflammation in AD. Inflammation and iron are ensnared within a self-reinforcing pathological feedback cycle: iron increases results in oxidative stress, which activates astrocytes and microglia, inducing immune cells to secrete pro-inflammatory cytokines and to sequester iron to attempt to control its toxicity.^55^ In AD, misfolded protein deposits (Aβ and tau tangles) trigger a long-term inflammatory reaction.^56^ Activated microglia tend to accumulate adjacent to plaques and become iron-loaded (due to consumption of metabolic waste and dysfunctional iron metabolism), creating local iron stores that manifest as QSM increases.^14^ Thus, an elevated QSM signal in a region, can be considered an indirect imaging marker of chronic neuroinflammation, reflecting iron-laden microglia and macrophages.^14^ While oxidative stress and ferroptosis represent plausible downstream consequences of dysregulated iron metabolism, these mechanisms remain speculative in the context of QSM, and our primary interpretation focuses on established links between iron accumulation and microglial activation.

We found that QSM values in multiple brain regions are correlated with inflammatory and immune response markers, including CSF IL-10RB, PD-L1, SCF, TWEAK, CSF-1, CXCL9, HGF, FLT3L, CD40, STAMPB, and IL-20, which emerged as top candidates. These markers reflect key pathways including microglial activation, immune checkpoint regulation, astrocyte and endothelial signaling, and peripheral immune cell recruitment. Their association with QSM suggests that peripheral immune dysregulation may be linked to regional iron accumulation and glial activity within the brain, reinforcing the role of neuroinflammation in early Alzheimer’s pathology. Our result suggests that regions of increased susceptibility are associated with inflammatory activity.^57^ Indeed, *post-mortem* AD brain studies are consistent with the notion and have found that regions of iron deposition overlap with microglial activation and elevated levels of pro-inflammatory cytokines.^13^ Consistent with this interpretation, regional QSM values showed significant correspondence with sTREM2 levels, further supporting the view that susceptibility increases reflect microglial-associated neuroimmune activity.

Importantly, QSM did not correlate with Aβ or tau biomarkers across modalities. This absence of association was observed not only for PET measures (where signal saturation can occur in symptomatic stages) but also for CSF and plasma p-tau181 and p-tau217, which do not exhibit ceiling effects. QSM may capture iron-related and neuroimmune processes that evolve independently of, or alongside, Aβ and tau pathology, particularly in later disease stages when protein aggregation may reach plateau,^58^ but glial and iron-handling alterations continue to progress. This interpretation is consistent with prior studies showing that QSM overlaps with Aβ deposition mainly in earlier or prodromal phases,^17,19^ while in more advanced stages its strongest signals correspond to susceptibility changes associated with inflammation and iron dysregulation.^4,59^ And notably, the spatial pattern of QSM elevation in our cohort closely followed the hierarchical organization of Braak-stage regions.

Although plasma inflammatory markers do not provide spatial specificity, they capture systemic immune signaling pathways that are increasingly recognized as modulators of central iron homeostasis and neuroimmune activation^60–62^ and therefore offer a biologically meaningful complement to imaging-based measures. Another imaging tool to measure neuroinflammatory changes in the AD brain is PET scanning of the 18 kDa translocator protein (TSPO), an upregulated marker present in activated microglia, has been employed to visualize neuroinflammation in AD. TSPO PET scans have shown increased radioligand binding in AD patients compared to healthy controls, with higher binding correlating with greater clinical impairment^63^ and pathology burden in some cohorts.^64^ However, the translational potential of TSPO imaging remains modest: there is no consensus across studies as to its pathophysiological sensitivity, some reporting low discrimination between AD and normal aging, and weak correlations with amyloid burden.^65^ Furthermore, PET imaging is expensive and not widely available, and genetic polymorphisms and off-target binding influence TSPO binding.^66^

In vivo studies compared QSM with PET tracers for microglial activation and observed that iron-rich areas generally coincide with regions of elevated TSPO uptake, relating susceptibility changes to inflammation in living patients.^14^ Consistent with prior multimodal work,^14,57,67^ TSPO-PET and iron-sensitive MRI show partial regional overlap (e.g. in limbic/association cortices) while also diverging in other areas because TSPO reflects microglial activation and QSM primarily indexes iron-related susceptibility, so the two signals need not always co-localize. The correlation of QSM with peripheral inflammatory markers may be explained via numerous mechanisms, including oxidative stress.^68^ In AD, inflammation and iron deposition would more than likely fuel each other over decades. QSM captures the iron component of this interaction and thereby serves as an intermediary between the molecular (iron, free radical damage) and cellular (microglial/astroglial activation) aspects of AD pathology. As a whole, QSM alterations are not isolated events but are linked with neuroinflammatory cascades—heightened susceptibility is a sign of areas under inflammatory siege, and conversely, inflammation may drive iron dysregulation. This renders QSM a useful surrogate for examining the inflammation aspect of AD and whether it may be responsible for causing neurodegeneration.

It is important to note that QSM does not directly image inflammation itself; rather, it reflects magnetic susceptibility changes that can result from iron-related processes associated with glial activation, oxidative stress, or cellular iron handling, and should therefore be interpreted as an indirect marker of neuroinflammatory activity. Although demyelination may influence QSM, especially in white matter, the predominantly cortical gray matter regions showing longitudinal susceptibility increases in our study contain relatively low myelin content, and their strong alignment with inflammation-related biomarker profiles supports the interpretation that iron-mediated microglial processes^13^ are the primary contributors to these changes.

A strength of this study is that all scans were acquired on the same 3 T system under routine phantom-based quality control, which reduces longitudinal variability and represents a recommended best practice for QSM studies. Beyond AD, QSM has also shown value in characterizing susceptibility changes during brain development and pediatric neurological conditions,^69–71^ highlighting its broader clinical applicability in detecting alterations in myelination and inflammation across the lifespan.

### Limitations

Future studies with longer data acquisition are required to validate our findings. As the iron deposition is a slow-paced phenomenon, longitudinal studies with longer follow-ups may capture a more precise distinction in Alzheimer’s disease-related regions. Participants in the TRIAD cohort are not representative of population diversity in North America, or indeed the world. This limits generalizability to other genetic backgrounds, lifestyle determinants, and environmental exposures in different populations. To minimize this limitation, in future research one needs to utilize cohorts of a more representative sample of racial and ethnic groups to enhance the generalizability of these results. Our study systematically excluded participants with comorbid conditions, particularly those with neurological disorders. Although this was necessary to limit confounding factors, it restricts the generalizability of our study’s results to patients with uncontrolled chronic diseases. Several neurodegenerative diseases are characterized by misfolded protein aggregation and potentially can impact the parameters investigated in our study. Subsequent studies would need to enroll patients with comorbid neurological illness to assess how these illnesses affect the biomarkers and imaging parameters evaluated in our research. It is important to note that QSM reflects a composite magnetic susceptibility signal arising from multiple tissue sources, and in this context, the observed associations with inflammatory biomarkers suggest that iron-related inflammatory processes are a likely contributor, while not excluding additional influences from myelin or other susceptibility sources. Finally, only the voxel susceptibility value in traditional QSM were analyzed here, which can be improved with finer signal modeling^72^ and which represents a sum of paramagnetic and diamagnetic sources.^73^ Recent techniques for separating paramagnetic (iron-related) from diamagnetic (myelin- or calcium-related) susceptibility components^74–76^ show promise for improving the specificity of QSM; however, these approaches remain under active methodological development and are not yet established for routine clinical or research use in AD.^77^ We acknowledge that the present study did not include a direct in vivo neuroinflammation imaging reference (e.g. TSPO–PET) or post-mortem validation, and therefore the interpretation of QSM as reflecting inflammation should be understood as indirect and based on its associations with immune-related biomarkers. Replication of these biomarker associations in an independent cohort will be important to confirm generalizability, although such validation was not feasible within the present dataset. Although all scans were acquired on a single 3 T system under routine phantom-based quality control, minor technical variability is inherent to longitudinal MRI measures, and QSM should be interpreted as an indirect marker of iron-related processes in the absence of a direct in vivo reference standard.

### Data availability

All requests for raw and analyzed data and materials will be promptly reviewed by McGill University to verify if the request is subject to any intellectual property or confidentiality obligations. Anonymized data will be shared upon request to the study’s senior author from a qualified academic investigator for the sole purpose of replicating the procedures and results presented in this article. Any data and materials that can be shared will be released via a material transfer agreement. Data are not publicly available due to the potential for information to compromise the privacy of research participants. Related documents, including study protocol and informed consent forms, can similarly be made available upon request.

## Conclusion

QSM detects paramagnetic tissue changes that correlate with inflammatory biomarker levels, but further validation against direct measures of neuroinflammation is necessary. We observed widespread regional QSM alterations, particularly in basal ganglia, limbic structures, and cortical gray matter. Our results support the notion that iron dysregulation may serve as a central pathological characteristic of AD, closely associated with inflammatory mechanisms. Notably, our longitudinal data highlighted the importance of at least 2 years of follow-up to capture disease-related susceptibility dysregulations in various brain regions. We found strong correlations between QSM and various inflammation markers, which pointed toward QSM being at the forefront MRI-based biomarker to capture AD progression and targeting iron and inflammation in therapeutic interventions.

## Supplemental Material

sj-docx-1-jcb-10.1177_0271678X261417193 – Supplemental material for Quantitative susceptibility mapping of the brain is associated with inflammatory changes in Alzheimer’s disease related areas Supplemental material, sj-docx-1-jcb-10.1177_0271678X261417193 for Quantitative susceptibility mapping of the brain is associated with inflammatory changes in Alzheimer’s disease related areas by Seyyed Ali Hosseini, Stijn Servaes, Arthur C Macedo, Etienne Aumont, Nesrine Rahmouni, Tevy Chan, Joseph Therriault, Lydia Trudel, Brandon Hall, Yi-Ting Wang, Jaime Fernandez Arias, Gleb Bezgin, Yansheng Zheng, Marina P Gonçalves, Kely Quispialaya Socualaya, Marcel S Woo, Cécile Tissot, Delphine Oliva-Lopez, Jieying Li, Stuart Mitchell, Aurélie Lebrun, Robert Hopewell, Sanjeev Chawla, Vladimir Fonov, Gassan Massarweh, Yasser Iturria Medina, Jean-Paul Soucy, Maxime Montembeault, Paolo Vitali, Kaj Blennow, Thomas K Karikari, Andréa L Benedet, Nicholas J Ashton, Henrik Zetterberg, Tharick A Pascoal, Serge Gauthier, Jesse Klostranec, Hangwei Zhuang, Junghun Cho, D Louis Collins, Yi Wang, David A Rudko and Pedro Rosa-Neto in Journal of Cerebral Blood Flow & Metabolism
