# Supplementary material for: Quantitative susceptibility mapping of the brain is associated with inflammatory changes in Alzheimer’s disease related areas
Source: J Cereb Blood Flow Metab. 2026 Feb 8:0271678X261417193. Online ahead of print. doi: 10.1177/0271678X261417193 (PMC12885976; doi:10.1177/0271678X261417193)
Supplement: sj-docx-1-jcb-10.1177_0271678X261417193 – Supplemental material for Quantitative susceptibility mapping of the brain is associated with inflammatory changes in Alzheimer’s disease related areas [file sj-docx-1-jcb-10.1177_0271678X261417193.docx]

**Quantitative Susceptibility Mapping of the Brain is Associated with Inflammatory Changes in Alzheimer’s Disease Related Areas**

Seyyed Ali Hosseini^1,2,3^, Stijn Servaes^1,2,3^, Arthur C Macedo^1,2,3^, Etienne Aumont^1,2,3^, Nesrine Rahmouni^1,2,3^, Tevy Chan^1,2,3^, Joseph Therriault^1,2,3^, Lydia Trudel^1,2,3^, Brandon Hall^1,2,3^, Yi‐Ting Wang^1,2,3^, Jaime Fernandez Arias^1,2,3^, Gleb Bezgin^1,2,3^, Yansheng Zheng^1,2,3^, Marina  P Gonçalves^1,2,3^, Kely Quispialaya Socualaya^1,2,3^, Marcel S Woo^1,4,5^, Cécile Tissot^6^, Delphine Oliva-Lopez^1,2,3^, Jieying Li^1^, Stuart Mitchell^1,2,3^, Aurélie Lebrun^1,2,3^, Robert Hopewell^1,3^, Sanjeev Chawla^7^, Vladimir Fonov^2^, Gassan Massarweh^2^, Yasser Iturria Medina^2^ , Jean-Paul Soucy^2^, Maxime Montembeault^3^, Paolo Vitali^2^,  Kaj Blennow^8,13,17,18^, Thomas K. Karikari^6,8,9,^, Andréa L. Benedet^8^, Nicholas J Ashton^8,10,11,12^, Henrik Zetterberg^8,13,14,15,19,20,21,22^, Tharick A Pascoal^23^, Serge Gauthier^2,3^, Jesse Klostranec^24^, Hangwei Zhuang^25^, Junghun Cho^25^, D. Louis Collins^2^ , Yi Wang^25^, David A. Rudko^2,16^ & Pedro Rosa‐Neto^1,2,3,26*^

**Supplementary Information, Section 1**

- - 1. **Plasma biomarkers**

Blood samples were collected and processed following previously described protocols (1). Plasma Aβ40 and Aβ42 were quantified using the **Simoa® (Single Molecule Array) platform (Quanterix).** Plasma p-tau181 and GFAP concentrations were measured using the **Simoa Neurology 3-Plex and HD-X assays (Quanterix),** and plasma NFL was assessed with the **Simoa NF-Light assay**. Plasma p-tau217 was measured using the **AlzPath assay**(2).

- - 1. **CSF biomarkers**

CSF Aβ40, Aβ42, total tau (t-tau), and p-tau181 were quantified using the **Lumipulse® G platform (Fujirebio)**. Additional p-tau isoforms, including p-tau217, p-tau231, p-tau202, and p-tau235, were measured using **Simoa-based immunoassays**. CSF NFL was determined using an **in-house validated ELISA**. Synaptic and glial biomarkers, such as neurogranin, SNAP-25, sTREM2, and YKL-40, were assessed with **Simoa, ELISA, or ECLIA assays**, based on platform availability (3, 4). All samples were processed and analyzed at the Clinical Neurochemistry Laboratory, University of Gothenburg, Sweden, with scientists blinded to clinical, demographic, and biomarker data.

- - 1. **Inflammatory biomarkers**

A panel of 92 inflammatory proteins was quantified in plasma using the **Olink® Target 96 Inflammation panel**, which applies **proximity extension assay (PEA) technology** to provide high-throughput, multiplexed protein detection with high sensitivity and specificity (5).

- - 1. **Mass spectrometry-based tau proteomics**

Site-specific tau phosphorylation was assessed using a **targeted liquid chromatography-tandem mass spectrometry (LC-MS/MS) workflow**. This approach quantified tau tryptic peptides covering key phosphorylation sites, including **p-tau181, p-tau202, p-tau217, p-tau231, and p-tau396**, enabling detailed characterization of tau phosphorylation stoichiometry across the disease spectrum.

**Supplementary Information, Section 2**

Study participants underwent amyloid-PET with [^18^F]AZD4694 and tau-PET with [^18^F]MK-6240 on the same brain-dedicated scanner (Siemens High-Resolution Research Tomograph, HRRT). [^18^F]AZD4694 images were acquired 40–70 minutes post-intravenous bolus injection and reconstructed using an ordered subset expectation maximization (OSEM) algorithm into a four-dimensional (4D) dataset comprising three frames (3 × 600 s). [^18^F]MK-6240 scans were obtained 90–110 minutes after tracer administration and reconstructed with the same OSEM algorithm into four frames (4 × 300 s), as previously reported (6, 7). Following each PET session, a 6-minute transmission scan using a rotating ^137^Cs point source was performed for attenuation correction. All PET data were corrected for motion, dead time, radioactive decay, and scattered and random coincidences. Images were first linearly aligned to the subject’s native T1-weighted MRI and then transformed to MNI standard space via a combination of linear and nonlinear registrations. PET data in native space were subsequently normalized to MNI space using transformations derived from the T1-weighted images and smoothed with an 8-mm full-width at half-maximum Gaussian kernel. Standardized uptake value ratios (SUVRs) for [^18^F]AZD4694 were computed using whole cerebellar gray matter as the reference region, whereas SUVRs for [^18^F]MK-6240 employed the inferior cerebellar gray matter reference.

**Supplementary references**

1. Karikari TK, Pascoal TA, Ashton NJ, Janelidze S, Benedet AL, Rodriguez JL, et al. Blood phosphorylated tau 181 as a biomarker for Alzheimer's disease: a diagnostic performance and prediction modelling study using data from four prospective cohorts. The Lancet Neurology. 2020;19(5):422-33.

2. Therriault J, Benedet AL, Pascoal TA, Mathotaarachchi S, Chamoun M, Savard M, et al. Association of apolipoprotein E ε4 with medial temporal tau independent of amyloid-β. JAMA neurology. 2020;77(4):470-9.

3. Therriault J, Vermeiren M, Servaes S, Tissot C, Ashton NJ, Benedet AL, et al. Association of phosphorylated tau biomarkers with amyloid positron emission tomography vs tau positron emission tomography. JAMA neurology. 2023;80(2):188-99.

4. Lantero‐Rodriguez J, Tissot C, Snellman A, Servaes S, Benedet AL, Rahmouni N, et al. Plasma and CSF concentrations of N‐terminal tau fragments associate with in vivo neurofibrillary tangle burden. Alzheimer's & Dementia. 2023;19(12):5343-54.

5. Pascoal TA, Benedet AL, Ashton NJ, Kang MS, Therriault J, Chamoun M, et al. Microglial activation and tau propagate jointly across Braak stages. Nature medicine. 2021;27(9):1592-9.

6. Therriault J, Benedet AL, Pascoal TA, Savard M, Ashton NJ, Chamoun M, et al. Determining amyloid-β positivity using 18F-AZD4694 PET imaging. Journal of Nuclear Medicine. 2021;62(2):247-52.

7. Jack Jr CR, Wiste HJ, Weigand SD, Therneau TM, Lowe VJ, Knopman DS, et al. Defining imaging biomarker cut points for brain aging and Alzheimer's disease. Alzheimer's & Dementia. 2017;13(3):205-16.

**
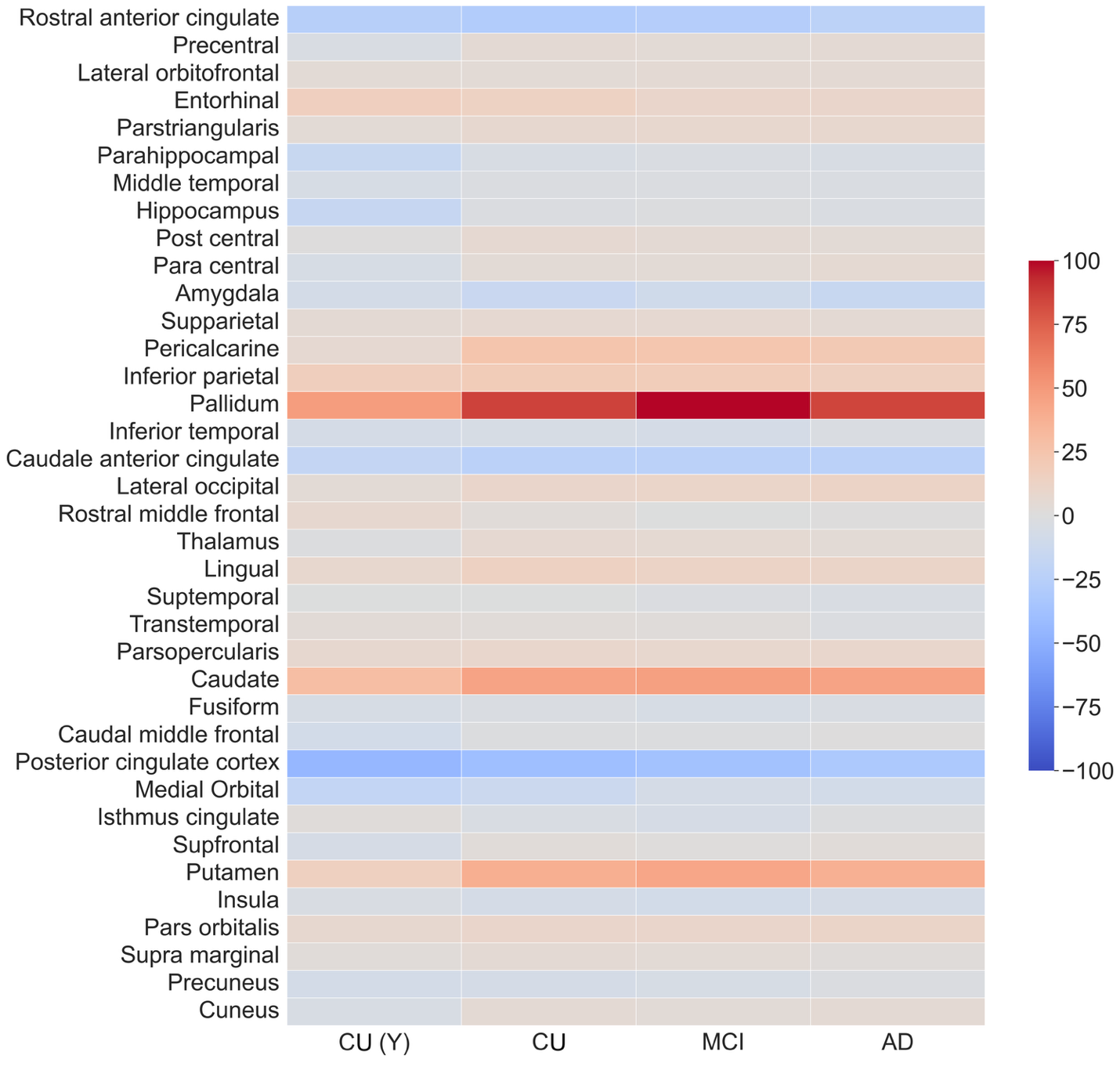
**

**Supplemental Figure1.** Heatmap of regional QSM values across cognitive groups, highlighting susceptibility differences in key brain regions.

**
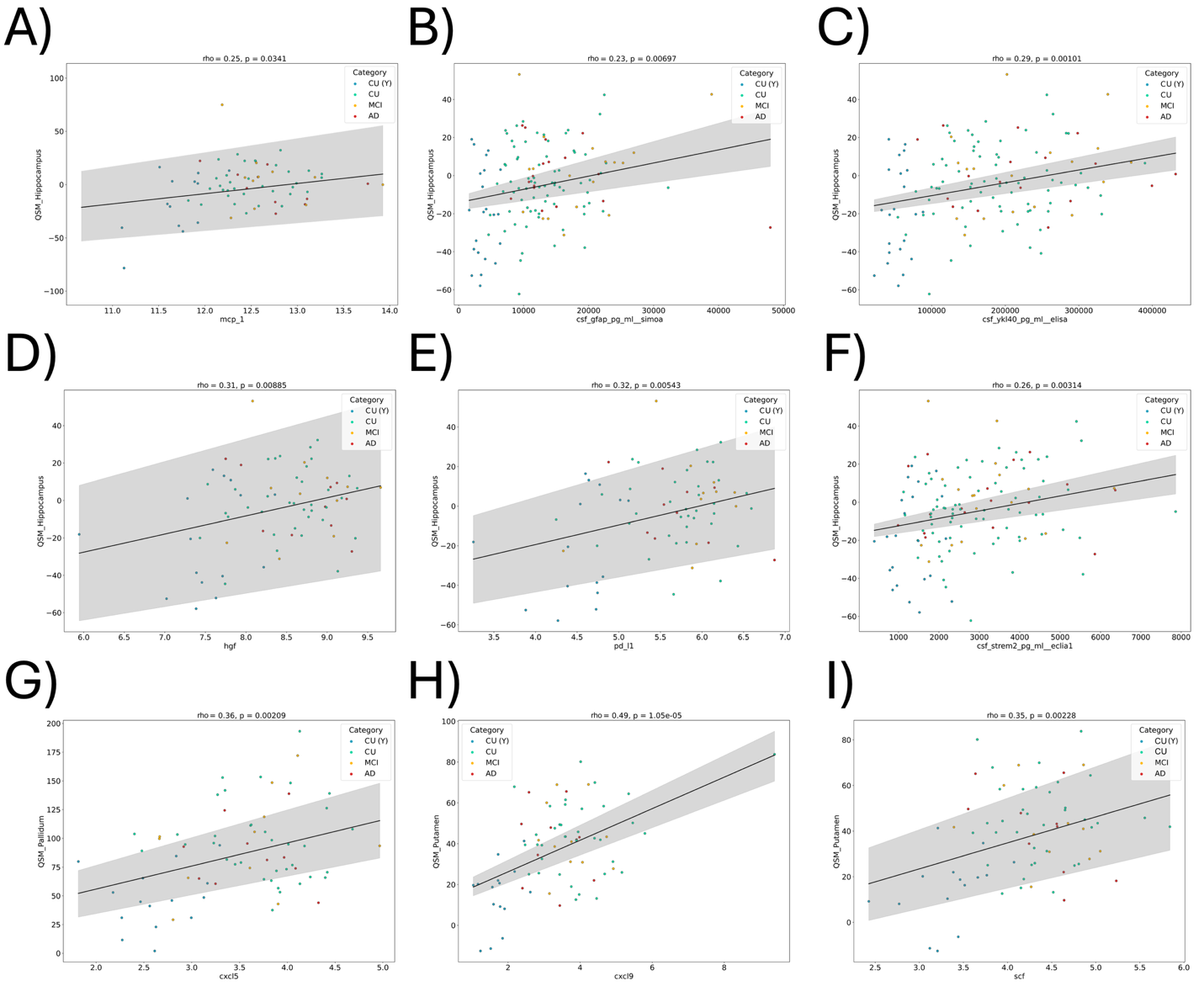
**

**Supplemental Figure2.** Scatter plots with linear regression lines, Spearman correlation coefficients, and p-values illustrate the relationships between significant correlated plasma and CSF inflammatory markers and QSM values across different brain regions.

**
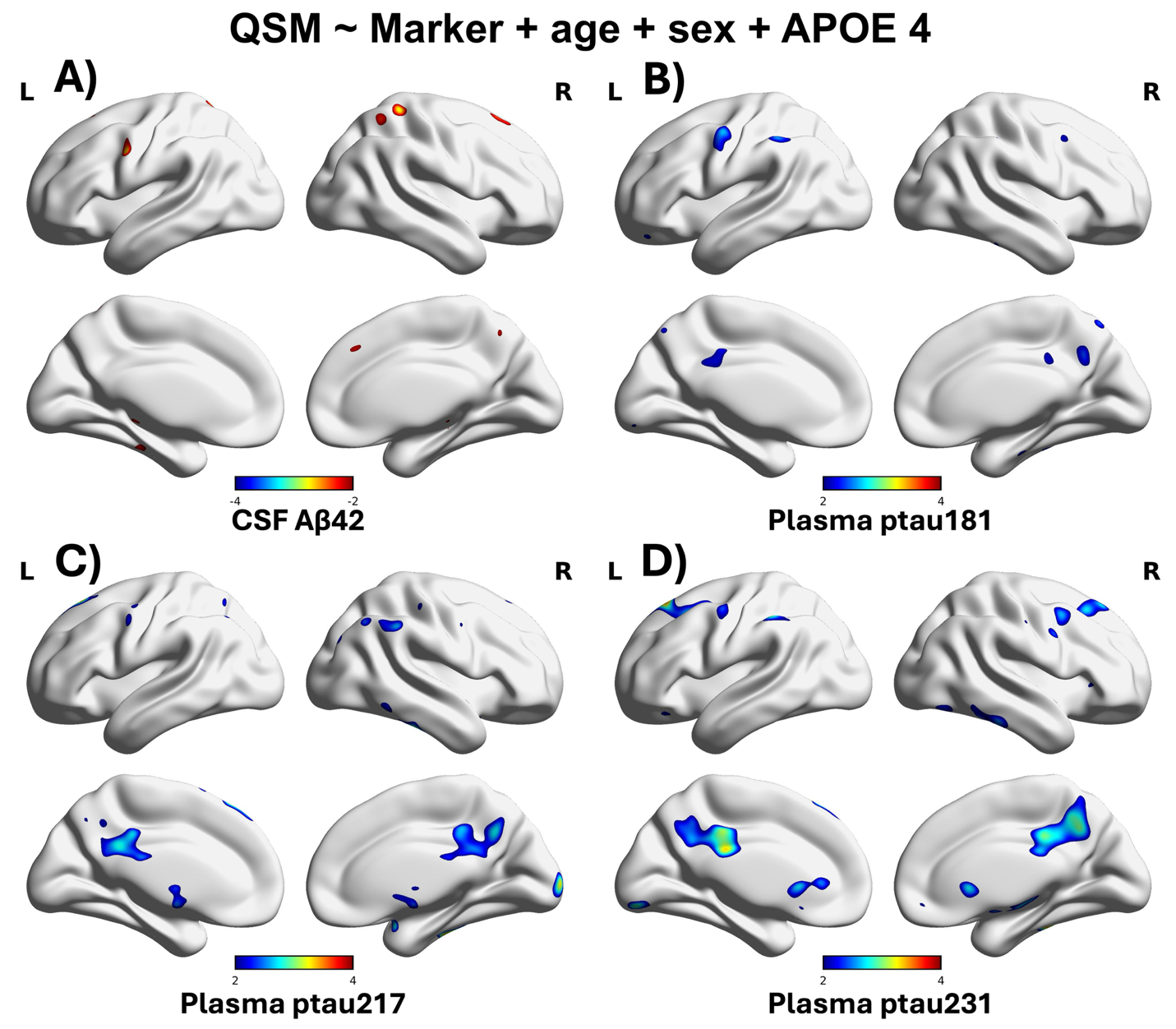
**

**Supplemental Figure3.** Voxel-based linear regression analyses revealed no significant associations between QSM maps and CSF Aβ40 or plasma p-tau isoforms (p-tau181, p-tau217, and p-tau231) in cortical regions. All models were adjusted for age, sex and APOE4, and multiple comparisons were corrected using random field theory (P < 0.001).
